# Synthesis and Cytotoxicity of Novel 10-Substituted Dihydroartemisinin Derivatives Containing *N*-Arylphenyl-ethenesulfonamide Groups

**DOI:** 10.3390/molecules18032864

**Published:** 2013-03-04

**Authors:** Yajing Liu, Zijian Liu, Jiyue Shi, Huimin Chen, Bin Mi, Peng Li, Ping Gong

**Affiliations:** Key Laboratory of Original New Drug Design and Discovery of Ministry of Education, School of Pharmaceutical Engineering, Shenyang Pharmaceutical University, Shenyang 110016, China

**Keywords:** 10-substituted dihydroartemisinin derivatives, synthesis, anti-cancer

## Abstract

The manuscript describes the synthesis of 10-substituted dihydroartemisinin derivatives containing *N*-aryl phenylethenesulfonamide groups and their *in vitro* anti-tumor activities against the HT-29, MDA-MB-231, U87MG, H460, A549 and HL-60 cancer cell lines and the normal WI-38 cell line. Most tested compounds showed enhanced cytotoxic activities and good selectivity toward the MDA-MB-231, HT-29 and HL-60 cell lines, with IC_50_ values in the single-digit μM range as compared with dihydroartemisinin (DHA), and all of them displayed less toxicity towards WI-38 cells. Among them, compounds **3c** and **6c** with trifluoromethoxy groups on the *N*-phenyl ring were found to be most active compounds against the six tested cancer cell lines.

## 1. Introduction

**Figure 1:**
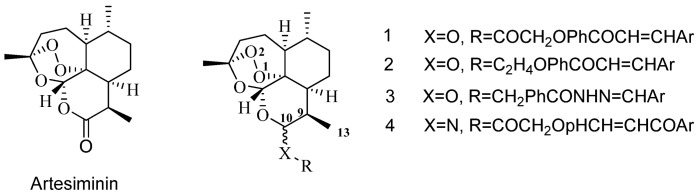
Structures of artesiminin derivatives.

Artemisinin is a sesquiterpene lactone isolated from the plant *Artemesia annua* L. Artemisinin and its derivatives, such as dihydroartemisinin (DHA) [1], artemether, arteether, and artesunate [2], are widely used currently as front-line antimalarials [3,4]. Despite the reported neurotoxic and embryotoxic effects in animals occurring at higher doses, application of artemisinins in humans seems to be relatively safe [5]. In addition to their well-known antimalarial activity, artemisinin derivatives possess cytotoxic activity against cancer cells by inducing apoptosis [6], but high concentrations are required [7]. Therefore, the synthesis of new, structurally modified derivatives of artemisinin is essential. The high chemical sensitivity of the artemisinin molecule restricts broad derivatization for library synthesis for further clinical development. So far the majority of the artemisinin derivatizations were carried out on the C-10 acetal and to a lesser extent on the C-13 carbon [8]. The observation that dihydroartemisinin C-10 ester, ether or amide derivatives (Figure 1) possess significant antitumor activity prompted previous efforts, both within our group [9,10] and by others [11,12,13].

**Figure 2:**
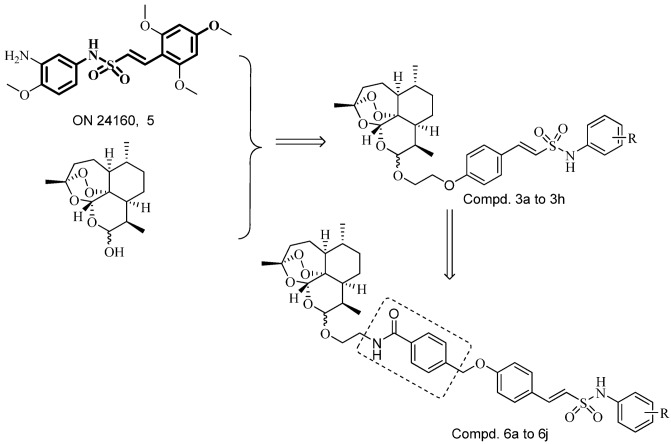
Design strategy for target compounds.

Hybrid drugs are formed by covalently linking two distinct chemical moieties with different biological modes of action with the aim of creating binary therapies with improved biological activity and less susceptible to the development of drug resistance [14,15]. Some *N*-aryl-2-arylethenesulfonamides, a new class of small molecule antitubulin agents, have exhibited potent activity against a wide spectrum of cancer cell lines, especially including some drug-resistant cell lines (e.g., MES-SA, HCT116, MDA-MB, *etc*.). Among them, **ON 24160** (**5**, Figure 2) was the most promising compound, with significant cytotoxicity against multiple cancer cell lines and a suitable pharmacokinetic profile [16,17]. Therefore, we have now synthesized a short series of dihydroartemisinin derivatives **3a**–**h** in which the C-10 hydroxy was covalently linked to the *N*-aryl-2-arylethenesulfonamide moiety with a two-carbon chain (Figure 2). To expand the search for new antitumor compounds, the *N*-methylbenzamide scaffold was next inserted into the two-carbon chain linker to obtain compounds **6a** to **6j** (Figure 2).

In this study, the synthesis of novel N-aryl phenylethenesulfonamides dihydroartemisinin derivatives (compounds **3a**–**h** and **6a**–**j**, Figure 2) was reported. Most of them exhibited potent cytotoxic activity against six human cancer cell lines, including the HT-29, MDA-MB-231, U87MG, H460, A549 and HL-60 cell lines.

## 2. Results and Discussion

### 2.1. Chemistry

**Scheme 1:**
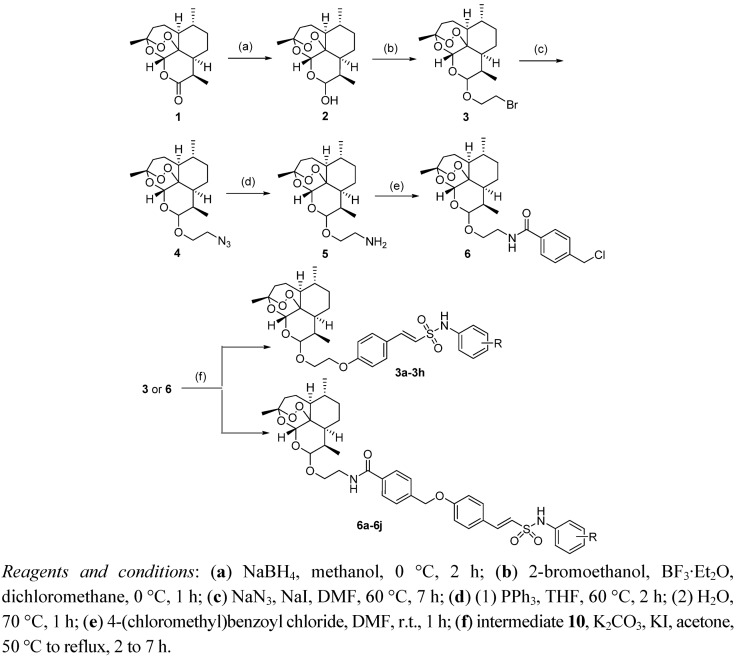
The synthetic route to the target compounds.

The syntheses of target compounds **3a**–**h** and **6a**–**j** are outlined in Scheme 1. Reduction of artemisinin (**1**) with NaBH_4_ gave dihydroartemisinin (**2**) as mixture of 10α and 10β epimers which reacted with 2-bromoethanol directly using the Lewis acid BF3·Et_2_O as catalyst to give 10-bromoethoxydihydro-artemisinin (**3**) [18]. In our previous report, the stereochemistry of compound **3** had already been determined as 10β by the ^1^H-NMR coupling constant (*J* = 3.4 Hz) between 9-H and 10-H and the chemical shift of 10-H (4.77 ppm) [9]. Subsequently, the target compounds **3a**–**h** were prepared in reasonable yield by potassium iodide-catalyzed substitution of bromo compound **3** with *N*-aryl-4-hydroxyphenylethane-sulfonamides **10** in the presence of potassium carbonate.

**Scheme 2:**
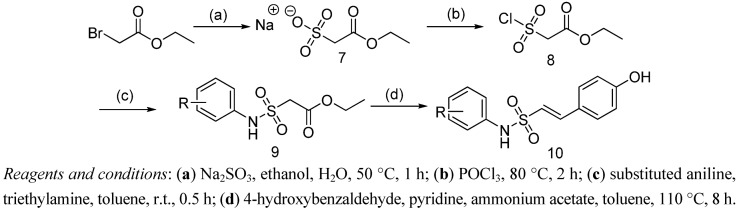
The synthetic route to the side chain **10**.

Regarding the side chain, *N*-aryl-2-arylethenesulfonamide derivatives **10** were prepared from ethyl bromoacetate according to Scheme 2. Reaction of ethyl bromoacetate with Na_2_SO_3_ afforded sodium 2-ethoxy-2-oxoethanesulfonate (**7**), which was converted to the sulfonyl chloride **8** via chlorination using POCl_3_. Then condensation of compound **8** with the corresponding substituted anilines in toluene gave the ethyl 2-(*N*-arylsulfamonyl)acetates **9**. Finally, reaction of compounds **9** with 4-hydroxybenzaldehyde afforded the desired side chains **10** in high yield.

The target compounds **6a**–**j** with the extend side chain were prepared from intermediate **3**. Firstly, treatment of **3** with sodium azide under sodium iodide catalysis in DMF according to a modified Haynes’ method [19] gave 10β-azidoethoxydihydroartemisinin (**4**). The Staudinger reduction was used in the transformation of azido compound **4** to 10β-aminoethoxydihydroartemisinin (**5**). Acylation of the amino group of **5** yielded 10β-arylamidoethoxydihydroartemisinin (**6**). Compounds **6a**–**j** were prepared using a procedure similar to that used to synthesize compounds **3a**–**h**.

The formation of the four key intermediates **3**–**6** was confirmed by ^1^H-NMR and LC-MS data, The formation of the title compounds **3a**–**h** and **6a**–**j** was evidenced by their ^1^H-NMR, IR and LC-MS spectra, explained in the Experimental section.

### 2.2. Biological Activity

The cytotoxic activities of target compounds were evaluated against HT-29 (human colon cancer), MDA-MB-231 (human breast cancer), U87MG (human glioblastoma), H460 (human lung cancer), A549 (non-small-cell lung adenocarcinoma), HL-60 (human leukemic) cancer cell lines and one normal cell line WI-38 (human fetal lung fibroblasts) together with references DHA and cisplatin by a MTT assay. The results, expressed as averaged IC_50_ values from at least three independent experiments, are summarized in Table 1.

As illustrated in Table 1, all of the target compounds showed moderate to excellent cytotoxic activities against the different cancer cell lines in the single-digit μM range, and displayed less toxicity against normal WI-38 cells. In general, most of the compounds displayed significant selectivity toward MDA-MB-231, HT-29 and HL-60 cancer cells. It was worth noting that compounds **3c** and 6c exhibited excellent anti-tumor activities against MDA-MB-231, HT-29 and HL-60 cells with IC_50_ values in the tenths of a digit nM range. Moreover, the selectivity index (IC_50_ normal cell/IC_50_ cancer cell) of compound **3c** for HL-60 was 750, which demonstrated that the tumor cells were more sensitive than the normal cells.

**Table 1:** Structures and cytotoxicity of compounds against HT-29, MDA-MB-231, U87MG, H460, A549, HL-60 and WI38 cell lines.

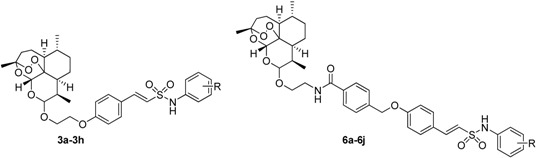								
Compd.	R	IC_50_(μM) ^a^						
		MDA	HT29	U87MG	H460	A549	HL-60	WI38
**3a**	H	2.00	0.68	3.97	5.38	2.16	1.25	16.75
**3b**	3-chloro	1.61	0.78	3.51	7.32	4.48	0.75	23.88
**3c**	4-trifluoromethoxy	0.39	0.20	1.59	0.97	2.11	0.05	37.47
**3d**	2-fluoro-5-methyl	1.27	1.78	5.71	4.29	3.64	0.84	20.53
**3e**	2-methyl	1.64	0.86	2.73	1.58	7.32	2.37	16.44
**3f**	4-chloro	1.04	0.42	0.79	1.41	3.52	0.08	19.88
**3g**	2-chloro	0.93	0.55	1.78	2.53	3.05	0.17	16.33
**3h**	2,6-dichloro	1.07	0.49	2.51	1.28	4.33	0.14	20.74
**6c**	4-trifluoromethoxy	0.49	0.29	3.71	2.70	1.57	0.09	28.17
**6b**	3-chloro	2.23	1.11	4.52	2.79	2.86	1.81	35.76
**6a**	H	2.26	0.98	6.47	5.28	3.21	2.41	20.04
**6d**	2-fluoro-5-methyl	1.33	1.82	17.24	10.88	9.72	1.30	19.28
**6e**	2-methyl	2.10	2.09	3.65	1.92	5.47	3.21	16.39
**6f**	4-chloro	2.38	0.92	1.59	1.77	5.72	1.33	24.70
**6i**	3-methoxy	8.89	8.95	>100	16.32	11.34	8.53	19.21
**6j**	4-fluoro-3-trifluoromethyl	1.05	0.38	3.27	5.31	2.78	1.27	30.37
**6g**	2-chloro	0.72	0.45	2.69	0.89	5.31	0.27	27.20
**6h**	2,6-dichloro	0.88	0.44	3.12	1.21	2.34	0.18	23.5
DHA ^b^		9.80	5.60	15.82	12.37	10.14	5.29	>100
Cisplatin ^b^		6.82	10.31	8.21	7.55	4.31	9.28	51.22

NA: compound showing IC_50_ value > 200 μM. ND: not determined. ^a^ IC_50_: concentration of the compound (μM) producing 50% cell growth inhibition after 72 h of drug exposure, as determined by the MTT assay. Each experiment was run at least three times, and the results are presented as average values ± standard deviation. ^b^ Used as a positive control.

Analysis of Table 1 clearly reveals that the cytotoxicities of benzamide derivatives **6a**–**j** were lower than those of the corresponding compounds **3a**–**h**. For example, the activity of compound **3c** was 2-, 3- and 2-fold higher than that of benzamide analog **6c** against U87MG, H460 and HL-60, respectively. In addition, it is worth mentioning that different biological properties were observed when a variety of groups were introduced into the *N*-phenyl moieties. The activities of compounds **3b**, **3d**, **3f**–**h**, **6b**, **6d** and **6f**–**h**, with the electron-withdrawing halide atoms on the *N*-phenyl moiety, were superior to those with electron-donating groups such as methoxy, or methyl groups or no substitutents (**6i**, **3e**, **6e**, **3a** and **6a**). In the first series of compounds (**3a**–**h**), the 4-chloro derivative (**3f**) was superior to the fluoro-substituted (**3d**), disubstituted (**3h**) or compounds substituted at other positions (**3b**,**g**). Nevertheless, the 2-chloro and 2,6-dichloro derivatives **6g**,**h** were better than others in the second series of compounds (**6a**–**j**). Excitingly, the compounds with a trifluoromethoxy group at the 4-position of the *N*-phenyl exhibited exceptionally high potency in two series of compounds, as exemplified by compounds **3c** and **6c**.

## 3. Experimental

### 3.1. General

Melting points were measured with a Büchi Melting Point B-540 apparatus (Büchi Labortechnik, Flawil, Switzerland) and are uncorrected. Mass spectra (MS) were taken in ESI mode on Agilent 1100 LC-MS (Agilent, Palo Alto, CA, USA). ^1^H-NMR spectra were performed using Bruker 300 MHz spectrometers (Bruker Bioscience, Billerica, MA, USA) with TMS as an internal standard. IR spectra (KBr disks) were recorded with a Bruker IFS 55 instrument (Bruker). Elemental analysis was determined on a Carlo-Erba 1106 Elemental analysis instrument (Carlo Erba, Milan, Italy). Unless otherwise noted, all the materials were obtained from commercially available sources and were used without further purification.

*Preparation of Dihydroartemisinin* (**2**). NaBH_4_ (12 g, 0.32 mol) was added slowly to a stirred solution of artemisinin (30 g, 0.11 mol) in methanol (300 mL) at −5~0 °C. The reaction mixture was stirred at 0 °C for 2 h, adjusted to pH 6 to 7 with acetic acid, and then concentrated under reduced pressure. The residue was poured into water (400 mL), and the solid product formed was collected by filtration, washed with water, and dried to yield compound **2** (25 g, 84%). m.p.: 145–148 °C; MS (ESI, *m/z*): 283.3 (M−H)^–^.

*Preparation of 2-(10β-Dihydroartemisinoxy)ethyl Bromide* (**3**). BF_3_^.^Et_2_O (16 mL) was added to a solution of dihydroartemisinin **2** (20 g, 70 mmol) and 2-bromoethanol (13 g, 0.11 mol) in CH_2_Cl_2_ (100 mL) below 0 °C. The mixture was stirred at room temperature until the reaction completed, and then was washed with saturated NaHCO_3_ solution, water and saturated NaCl solution. The organic layer was dried and concentrated. The residue was recrystallized from petroleum ether to give compound **3** (13 g, 46%) as white crystals. m.p.: 155–158 °C; MS (ESI, *m/z*): 413.2 (M+Na)^+^; ^1^H-NMR (300 MHz, DMSO-*d_6_*) δ: 5.43 (s, 1H), 4.77 (d, *J* = 3.4 Hz, 1H), 3.94 (td, *J* = 9.3, 4.1 Hz, 1H), 3.78 (m, 3H), 2.47 (m, 1H), 2.25 (m, 1H), 2.05 (m, 1H), 1.89–1.75 (m, 2H), 1.61 (m, 2H), 1.44–1.31 (m, 3H), 1.29 (s, 3H), 1.22–1.06 (m, 2H), 0.90 (d, *J* = 6.5 Hz, 3H), 0.87 (d, *J* = 7.5 Hz, 3H).

*Preparation of 2-(10β-Dihydroartemisinoxy)ethyl Azide* (**4**). NaN_3_ (4.0 g, 60 mmol) was added to a stirred solution of compound **3** (8.0 g, 21 mmol) and sodium iodide (0.15 g, 1 mmol) in DMF (60 mL). The reaction mixture was heated to 60 °C for 3–5 h. The mixture was poured into ice water, stirred for 1 h and separated by filtration to give white compound **4** (7.1 g, 98%). m.p.: 86–88 °C; MS (ESI, *m/z*): 376.2 (M+Na)^+^; ^1^H-NMR (300 MHz, DMSO-*d_6_*) δ: 5.39 (s, 1H), 4.76 (d, *J* = 3.3 Hz, 1H), 3.91 (m, 1H), 3.56 (m, 3H), 2.47 (m, 1H), 2.25 (m, 1H), 2.06 (m, 1H), 1.87 (m, 4H), 1.45 (m, 3H), 1.29(s, 3H), 1.14 (m, 2H), 0.90 (d, *J* = 3.8 Hz, 3H), 0.88 (d, *J* = 4.9 Hz, 3H).

*Preparation of 2-(10β-Dihydroartemisinoxy)ethylamine* (**5**). A 100 mL round-bottomed flask was charged with compound **4** (6.0 g, 17 mmol) and THF (60 mL). To this solution, triphenylphosphine (7.2 g, 27 mmol) was added slowly, and the reaction mixture was stirred for 2 h at 60 °C. Several drops of water (1.0 mL, 56 mmol) were added, and the resulting suspension was stirred for 6 h. The mixture was concentrated under reduced pressure. The crude mixture was purified by flash column chromatography (silica gel, dichloromethane/methanol, 200:1) to afford the desired compound **5** (4.1 g, 74%) as yellow oil. MS (ESI, *m/z*): 328.1 (M+H)^+^; ^1^H-NMR (300 MHz, DMSO-*d_6_*) δ: 5.34 (s, 1H), 4.69 (d, *J* = 3.4 Hz, 1H), 3.71 (m, 1H), 3.30 (dt, *J* = 9.8, 5.7 Hz, 1H), 2.73 (m, 2H), 2.47 (m, 1H), 2.25 (m, 1H), 2.07 (m, 1H), 1.86 (m, 2H), 1.78 (m, 2H), 1.63 (m, 3H), 1.29 (s, 3H), 1.21–1.07 (m, 2H), 0.89 (d, *J* = 6.3 Hz, 3H), 0.85 (d, *J* = 7.4 Hz, 3H).

*Preparation of 4-(Chloromethyl)-N-2-(10β-dihydroartemisinoxy)ethyl Benzamide* (**6**). A solution of compound **5** (4.0 g, 12 mmol) in DMF (20 mL) was cooled below 0 °C and 4-(chloromethyl)benzoyl chloride (2.3 g, 12 mmol) was added dropwise. The mixture was stirred at room temperature for 1 h, and then poured into water. The white precipitate was filtered, washed with ethanol, and dried to obtain **6** (5.1 g, 87%). m.p.: 105–108 °C; MS (ESI, *m/z*): 480.9 (M+H)^+^; ^1^H-NMR (300 MHz, DMSO-*d_6_*) δ: 7.93 (d, *J* = 7.2 Hz, 2H), 7.51 (d, *J* = 7.2 Hz, 2H), 5.38 (s, 1H), 4.71 (d, *J* = 3.4 Hz, 1H), 4.54 (s, 2H), 3.73 (m, 1H), 3.32 (dt, *J* = 9.8, 5.7 Hz, 1H), 2.75 (m, 2H), 2.49 (m, 1H), 2.28 (m, 1H), 2.11 (m, 1H), 1.89 (m, 2H), 1.80 (m, 2H), 1.65 (m, 3H), 1.31 (s, 3H), 1.24 (m, 2H), 0.92 (d, *J* = 6.4 Hz, 3H), 0.87 (d, *J* = 7.4 Hz, 3H).

*Preparation of Sodium 2-Ethoxy-2-oxoethanesulfonate* (**7**). Na_2_SO_3_ (15 g, 0.12 mol) was dissolved in water (50 mL) and a solution of ethyl bromoacetate (20 g, 0.12 mol) in ethanol (25 mL) was added dropwise at 5–10 °C. The reaction mixture was heated to 50 °C for 1 h. After then, the solution was evaporated until dryness. Acetic acid (200 mL) and ethyl acetate (100 mL) were added to the residue, and the mixture was heated to 100 °C for 1 h. The hot mixture was filtered, and another 1,000 mL ethyl acetate was poured into the filtrate. The white crystals were filtered, washed with ethyl acetate, and dried to yield white solid **7** (23 g, 99%). m.p.: 158 °C (dec.); MS (ESI, *m/z*): 190.2 (M+Na)^+^.

*Preparation of Ethyl 2-(Chlorosulfonyl)acetate* (**8**) A mixture of compound **7** (10 g, 0.053 mol) and POCl_3_ (100 mL, 1.1 mol) was stirred at 80 °C for 2 h and then cooled to room temperature. The solvent was removed under vacuum, 100 mL of toluene was added, and stirred for 10 min. The reaction solution was filtered, and the solvent was evaporated under reduced pressure to obtain **8** (9.0 g, 92%) as a red oil.

### 3.2. General Procedure for the Preparation of Ethyl 2-(N-Arylsulfamoyl)acetate *(**9**)*

A solution of **8** (8.0 g, 0.043 mol) in toluene (100 mL) was cooled to −5~0 °C, a mixture of substituted aniline (0.086 mol), triethylamine (0.065 mol) in toluene (50 mL) was added dropwise. The reaction mixture was stirred at room temperature for 0.5–1 h, and then washed with water, 5% HCl, saturated NaHCO_3_ solution, saturated NaCl solution and water. The organic phase was dried with Na_2_SO_4_ and evaporated to yield **9** as a brown oil.

### 3.3. General Procedure for the Preparation of Ethyl 2-(4-Hydroxyphenyl)-N-arylethenesulfonamides ***10***

A mixture of compound **9** (0.02 mol), 4-hydroxybenzaldehyde (5.0 g, 0.04 mol), pyridine (0.40 mL, 5.0 mmol), ammonium acetate (0.40 g, 5.0 mmol) and toluene (100 mL) was stirred at 110 °C for 8–10 h and then cooled to room temperature. The toluene phase was washed with 5% HCl, saturated NaHCO_3_ solution and water, dried with Na_2_SO_4_ to obtain **10** as a brick red oil.

### 3.4. General Procedure for the Preparation of Target Compounds ***3a**–**h***

A mixture of compound 3 (10 g, 0.026 mol), 10 (0.026 mol), K_2_CO_3_ (5.4 g, 0.039 mol), KI (0.40 g, 2.6 mmol) and acetone (100 mL) was stirred at 50 °C for 2–3 h. The reaction solvent was evaporated and the residue was purified by column chromatography (silica-gel, 1%–5% petroleum ether/ethyl acetate) to afford pure compounds **3a**–**h**.

### 3.5. General Procedure for the Preparation of Target Compounds ***6a**–**j***

A mixture of compound **10** (0.024 mol), K_2_CO_3_ (4.2 g, 0.03 mol) and acetone (100 mL) was stirred at room temperature for 10 min, and then compound 6 (9.6 g, 0.02 mol) and KI (0.4 g, 2.6 mmol) was added while the reaction refluxed for 7–9 h. The acetone was removed under vacuum, and CH_2_Cl_2_ (100 mL) was poured into the residue. The solution was washed with water, dried with Na_2_SO_4_. The solvent was evaporated and the residue was purified by column chromatography (silica-gel, 1%–5% petroleum ether/ethyl acetate) to afford pure compounds **6a**–**j**.

*(E)-2-(4-(2-(10β-Dihydroartemisinoxy)ethoxy)phenyl)-N-phenylethenesulfonamide* (**3a**). Light yellow solid (38% yield); m.p.: 136–138 °C; MS (ESI) *m/z*: 584.2 (M-H)^–^; IR (KBr) cm^−1^: 3422.1, 2923.1, 1603.8, 1514.4, 1343.4, 1147.5, 1027.3, 983.5, 696.9, 597.1; ^1^H-NMR (300 MHz, DMSO-*d*_6_) *δ*: 7.54 (d, *J* = 7.2 Hz, 2H), 7.35 (m, 5H), 7.15 (d, *J* = 15.2 Hz, 1H), 7.05 (d, *J* = 15.2 Hz, 1H), 6.79 (d, *J* = 7.5 Hz, 2H), 5.22 (s, 1H), 4.60 (s, 1H), 3.80 (m, 2H), 3.70 (m, 2H), 3.28 (m, 1H), 2.83 (m, 2H), 2.49 (m, 1H), 2.33 (m, 1H), 2.16 (m, 1H), 1.99 (m, 1H), 1.76 (m, 1H), 1.47 (m, 1H), 1.28 (m, 2H), 1.26 (s, 3H), 1.08 (s, 1H), 0.88 (d, *J* = 4.8 Hz, 3H), 0.75 (d, *J* = 7.1 Hz, 3H). Anal. Calcd. for C_31_H_39_NO_8_S: C 63.57, H 6.71, N 2.39. Found: C 63.52, H 6.50, N 2.41.

*(E)-2-(4-(2-(10β-Dihydroartemisinoxy)ethoxy)phenyl)-N-(3-chlorophenyl)ethenesulfonamide* (**3b**). Light yellow solid (44% yield); m.p.: 137–138 °C; MS (ESI) *m/z*: 618.0 (M−H)^–^; IR (KBr) cm^−1^: 3477.3, 2923.4, 1603.4, 1514.3, 1343.9, 1148.9, 1027.6, 983.4, 788.6, 597.7; ^1^H-NMR (300 MHz, DMSO-*d*_6_) *δ* 10.12(br, 1H), 7.58 (d, *J* = 8.6 Hz, 2H), 7.45 (m, 4H), 7.19 (d, *J* = 15.3 Hz, 1H), 7.08 (d, *J* = 15.3 Hz, 1H), 6.80 (d, *J* = 8.6 Hz, 2H), 5.22 (s, 1H), 4.60 (d, *J* = 3.1 Hz, 1H), 3.83 (m, 2H), 3.71 (m, 2H), 3.04 (s, 1H), 2.32 (m, 2H), 2.13 (d, *J* = 10.0 Hz, 1H), 2.00 (t, *J* = 13.9 Hz, 1H), 1.76 (m, 1H), 1.45 (m, 2H), 1.25 (d, *J* = 8.7 Hz, 5H), 1.11 (d, *J* = 18.8 Hz, 2H), 0.88 (d, *J* = 6.2 Hz, 3H), 0.72 (d, *J* = 7.4 Hz, 3H). Anal. Calcd. for C_31_H_38_ClNO_8_S: C 60.04, H 6.18, N 2.26. Found: C 59.98, H 6.21, N 2.19.

*(E)-2-(4-(2-(10β-Dihydroartemisinoxy)ethoxy)phenyl)-N-(4-trifluoromethoxyphenyl)ethenesulfonamide* (**3c**). Yellow solid (42% yield); m.p.: 139–142 °C; MS (ESI) *m/z*: 692.1 (M+Na)^+^; IR (KBr) cm^−1^: 3416.7, 2924.6, 1604.1, 1510.7, 1346.0, 1258.8, 1224.3, 1027.9, 983.6, 838.8, 504.3; ^1^H-NMR (300 MHz, DMSO-*d*_6_) *δ* 10.06 (br, 1H), 7.56 (d, *J* = 8.5 Hz, 2H), 7.48 (d, *J* = 8.9 Hz, 2H), 7.37 (d, *J* = 8.5 Hz, 2H), 7.19 (d, *J* = 15.3 Hz, 1H), 7.08 (d, *J* = 15.3 Hz, 1H), 6.79 (d, *J* = 8.6 Hz, 2H), 5.21 (s, 1H), 4.60 (d, *J* = 3.3 Hz, 1H), 3.82 (m, 2H), 3.76 (m, 2H), 2.32 (m, 1H), 2.15 (m, 1H), 2.00 (m, 1H), 1.76 (m, 1H), 1.46 (m, 2H), 1.41 (m, 1H), 1.26 (m, 6H), 1.09 (m, 2H), 0.87 (m, 3H), 0.70 (d, *J* = 7.3 Hz, 3H). Anal. Calcd. for C_32_H_38_F_3_NO_9_S: C 57.39, H 5.72, N 2.09. Found: C 57.41, H 5.68, N 2.11.

*(E)-2-(4-(2-(10β-Dihydroartemisinoxy)ethoxy)phenyl)-N-(2-fluoro-5-methylphenyl)ethenesulfonamide* (**3d**). Light yellow solid (36% yield); m.p.: 138–140 °C; MS (ESI) *m/z*: 640.1 (M+Na)^+^; IR (KBr) cm^−1^: 3424.5, 2923.5, 1604.1, 1510.7, 1343.8, 1148.7, 1028.1, 984.6, 872.5, 601.1; ^1^H-NMR (300 MHz, DMSO-*d*_6_) *δ* 10.06 (br, 1H), 7.56 (d, *J* = 8.5 Hz, 2H), 7.24 (m, 2H), 7.18 (m, 2H), 7.09 (d, *J* = 15.3 Hz, 1H), 6.81 (d, *J* = 8.6 Hz, 2H), 5.22 (s, 1H), 4.59 (d, *J* = 3.2 Hz, 1H), 3.72 (m, 4H), 2.26 (m, 1H), 2.15 (s, 3H), 2.10 (m, 1H), 2.00 (m, 1H), 1.77 (m, 1H), 1.40 (m, 3H), 1.28 (m, 6H), 1.09 (m, 2H), 0.87 (d, *J* = 5.9 Hz, 3H), 0.71 (d, *J* = 7.3 Hz, 3H). Anal. Calcd. for C_32_H_40_FNO_8_S: C 62.22, H 6.53, N 2.27. Found: C 62.19, H 6.55, N 2.29.

*(E)-2-(4-(2-(10β-Dihydroartemisinoxy)ethoxy)phenyl)-N-(2-methylphenyl)ethenesulfonamide* (**3e**). Light brown solid (26% yield); m.p.: 136–138 °C; MS (ESI) *m/z*: 598.2 (M−H)^–^; IR (KBr) cm^−1^: 3414.8, 2923.1, 1604.2, 1514.7, 1342.5, 1146.2, 1027.5, 983.6, 872.4, 603.3; ^1^H-NMR (300 MHz, DMSO-*d*_6_) *δ* 7.59 (d, *J* = 8.5 Hz, 2H), 7.28 (m, 6H), 6.80 (d, *J* = 8.6 Hz, 2H), 5.15(s, 1H), 4.62 (d, *J* = 3.3 Hz, 1H), 4.45 (d, *J* = 5.7 Hz, 1H), 3.82 (m, 2H), 3.70 (m, 2H), 2.35 (s, 3H), 2.07–1.87 (m, 1H), 1.75 (m, 1H), 1.42 (m, 2H), 1.26 (s, 3H), 1.25 (m, 4H), 1.06 (t, *J* = 7.0 Hz, 2H), 0.86 (d, *J* = 5.9 Hz, 3H), 0.73 (d, *J* = 7.3 Hz, 3H). Anal. Calcd. for C_32_H_41_NO_8_S: C 64.09, H 6.89, N 2.34. Found: C 64.13, H 6.81, N 2.39.

*(E)-2-(4-(2-(10β-Dihydroartemisinoxy)ethoxy)phenyl)-N-(4-chlorophenyl)ethenesulfonamide* (**3f**). Light brown solid (44% yield); m.p.: 138–141 °C; MS (ESI) *m/z*: 618.1 (M−H)^–^; IR (KBr) cm^−1^: 3411.5, 2925.1, 1601.5, 1490.3, 1343.2, 1149.3, 1054.2, 1027.0, 1007.0, 822.9, 760.8; ^1^H-NMR (300 MHz, DMSO-*d*_6_) *δ* 7.56 (d, *J* = 8.3 Hz, 1H), 7.43 (m, 3H), 7.18 (m, 2H), 7.01 (d, *J* = 8.8 Hz, 1H), 6.80 (d, *J* = 7.6 Hz, 1H), 6.67 (d, *J* = 7.0 Hz, 1H), 6.50 (d, *J* = 9.0 Hz, 1H), 5.17 (s, 1H), 4.76 (d, *J* = 3.5 Hz, 1H), 4.35 (m, 2H), 3.82 (m, 2H), 3.62 (m, 2H), 2.05 (s, 3H), 1.56 (m, 2H), 1.35 (m, 2H), 1.24 (s, 3H), 1.10 (m, 3H), 0.97 (d, *J* = 6.0 Hz, 3H), 0.80 (d, *J* =7.4 Hz, 3H). Anal. Calcd. for C_31_H_38_ClNO_8_S: C 60.04, H 6.18, N 2.26. Found: C 60.11, H 6.12, N 2.30.

*(E)-2-(4-(2-(10β-Dihydroartemisinoxy)ethoxy)phenyl)-N-(2-chlorophenyl)ethenesulfonamide* (**3g**). Yellow solid (38% yield); m.p.: 141–143 °C; MS (ESI) *m/z*: 618.2 (M-H)^–^; IR (KBr) cm^−1^: 3412.1, 2926.3, 1604.2, 1493.5, 1340.3, 1145.7, 1052.8, 1022.4, 828.5, 772.4; ^1^H-NMR (300 MHz, DMSO-*d*_6_) *δ* 7.65 (d, *J* = 8.2 Hz, 2H), 7.45 (m, 2H), 7.28 (m, 2H), 7.20 (d, *J* = 15.1 Hz, 1H), 7.04 (d, *J* = 15.1 Hz, 1H), 6.81 (d, *J* = 8.3 Hz, 2H), 5.27 (s, 1H), 4.62 (d, *J* = 3.2 Hz, 1H), 3.82 (m, 2H), 3.76 (m, 2H), 3.30 (m, 2H), 2.81 (m, 1H), 2.53 (m, 1H), 2.37 (m, 1H), 2.20 (m, 1H), 2.00 (m, 1H), 1.85 (m, 1H), 1.51 (m, 1H), 1.32 (m, 2H), 1.27 (s, 3H), 1.09 (s, 1H), 0.85 (d, *J* = 6.8 Hz, 3H), 0.79 (d, *J* = 7.3 Hz, 3H). Anal. Calcd. for C_31_H_38_ClNO_8_S: C 60.04, H 6.18, N 2.26. Found: C 59.93, H 6.21, N 2.29.

*(E)-2-(4-(2-(10β-Dihydroartemisinoxy)ethoxy)phenyl)-N-(2,6-dichlorophenyl)ethenesulfonamide* (**3h**). Brown solid (41% yield); m.p.: 144–146 °C; MS (ESI) *m/z*: 653.1 (M-H)^–^; IR (KBr) cm^−1^: 3420.8, 2922.6, 1602.6, 1508.2, 1348.2, 1147.1, 1027.8, 984.7, 874.3, 541.5; ^1^H-NMR (300 MHz, DMSO-*d*_6_) *δ* 7.78 (d, *J* = 8.1 Hz, 2H), 7.28 (m, 2H), 7.15 (d, *J* = 15.3 Hz, 1H), 6.97 (m, 2H), 6.83 (d, *J* = 7.9 Hz, 2H), 5.21 (s, 1H), 4.62 (d, *J* = 3.3 Hz, 1H), 3.82 (m, 4H), 2.31 (m, 2H), 2.16 (s, 2H), 2.12 (m, 1H), 1.98 (m, 1H), 1.75 (m, 2H), 1.39 (m, 2H), 1.26 (m, 6H), 1.07 (m, 2H), 0.89 (d, *J* = 5.9 Hz, 3H), 0.78 (d, *J* = 7.4 Hz, 3H). Anal. Calcd. for C_31_H_37_Cl_2_NO_8_S: C 56.88, H 5.70, N 2.14. Found: C 56.92, H 5.75, N 2.17.

*(E)-N-(2-(10β-Dihydroartemisinoxy)ethyl)-4((4-(2-(N-phenylsulfamoyl)vinyl)phenoxy)methyl)benzamide* (**6a**). Light yellow solid (48% yield); m.p.: 142–145 °C; MS (ESI) *m/z*: 717.1 (M-H)^–^; IR (KBr) cm^−1^: 3413.1, 2924.1, 1604.2, 1513.9, 1336.6, 1146.1, 1020.8, 873.7, 801.7, 699.4, 603.3; ^1^H-NMR (300 MHz, DMSO-*d*_6_) *δ* 8.49 (br, 1H), 7.85 (d, *J* = 8.1 Hz, 2H), 7.62 (m, 2H), 7.50 (d, *J* = 8.0 Hz, 2H), 7.33 (d, *J* = 15.2 Hz, 1H), 7.24 (t, *J* = 7.6 Hz, 2H), 7.17 (d, *J* = 8.1 Hz, 2H), 7.0 (m, 4H), 5.26 (s, 1H), 5.21 (s, 2H), 4.69 (d, *J* = 3.2 Hz, 1H), 4.44 (d, *J* = 5.2 Hz, 1H), 4.39 (t, *J* = 3.9 Hz, 2H), 3.82 (m, 2H), 2.24 (m, 2H), 2.06 (m, 3H), 1.67 (m, 3H), 1.48 (m, 2H), 1.26 (s, 3H), 1.23 (s, 1H), 0.81 (d, *J* = 7.4 Hz, 3H), 0.68 (d, *J* = 5.6 Hz, 3H). Anal. Calcd. for C_39_H_46_N_2_O_9_S: C 65.16, H 6.45, N 3.90. Found: C 65.09, H 6.43, N 3.96.

*(E)-N-(2-(10β-Dihydroartemisinoxy)ethyl)-4((4-(2-(N-(3-chlorophenyl)sulfamoyl)vinyl)phenoxy)methyl)benzamide* (**6b**). Light yellow solid (45% yield); m.p.: 146–149 °C; MS (ESI) *m/z*: 751.0 (M-H)^–^; IR (KBr) cm^−1^: 3397.3, 2923.3, 1602.5, 1512.7, 1344.3, 1147.4, 1026.6, 983.7, 872.9, 791.7, 594.8; ^1^H-NMR (300 MHz, DMSO-*d*_6_) *δ* 8.40 (br, 1H), 7.74 (d, *J* = 8.2 Hz, 2H), 7.60 (d, *J* = 8.5 Hz, 2H), 7.50 (d, *J* = 13.1 Hz, 2H), 7.37 (d, *J* = 8.2 Hz, 2H), 7.32 (d, *J* = 4.6 Hz, 2H), 7.26 (d, *J* = 8.1 Hz, 2H), 6.82 (d, *J* = 8.2 Hz, 2H), 5.25 (s, 1H), 4.92 (d, *J* = 13.4 Hz, 2H), 4.68 (d, *J* = 3.0 Hz, 1H), 3.82 (m, 2H), 3.53(m, 2H), 2.34 (m, 1H), 2.12 (m, 2H), 2.00 (m, 2H), 1.66 (m, 2H), 1.49 (m, 2H), 1.26 (m, 6H), 0.80 (d, *J* = 7.2 Hz, 3H), 0.65 (d, *J* = 5.7 Hz, 3H). Anal. Calcd. for C_39_H_45_ClN_2_O_9_S: C 62.18, H 6.02, N 3.72. Found: C 62.23, H 6.18, N 3.68.

*(E)-N-(2-(10β-Dihydroartemisinoxy)ethyl)-4((4-(2-(N-(4-trifluoromethoxyphenyl)sulfamoyl)vinyl)phenoxy)methyl)benzamide* (**6c**). Yellow solid (43% yield); m.p.: 149–151 °C; MS (ESI) *m/z*: 801.2 (M−H)^–^; IR (KBr) cm^−1^: 3396.9, 2924.0, 1603.0, 1507.9, 1346.4, 1360.2, 1146.2, 1025.4, 870.5, 801.6, 593.1; ^1^H-NMR (300 MHz, DMSO-*d*_6_) *δ* 8.38 (br, 1H), 7.74 (d, *J* = 8.2 Hz, 2H), 7.60 (d, *J* = 8.5 Hz, 2H), 7.47 (d, *J* = 9.0 Hz, 2H), 7.37 (d, *J* = 8.0 Hz, 2H), 7.34 (m, 4H), 6.82 (d, *J* = 8.2 Hz, 2H), 5.25 (s, 1H), 4.88 (s, 2H), 4.68 (d, *J* = 3.2 Hz, 1H), 4.45 (m, 1H), 4.35 (m, 1H), 3.82 (s, 2H), 3.62 (m, 1H), 2.35 (m, 1H), 2.11 (m, 1H), 1.96 (m, 1H), 1.65 (m, 3H), 1.51 (m, 1H), 1.25 (m, 7H), 0.79 (d, *J* = 7.2 Hz, 3H), 0.65 (d, *J* = 5.8 Hz, 3H). Anal. Calcd. for C_40_H_45_F_3_N_2_O_10_S: C 59.84, H 5.65, N 3.49. Found: C 59.93, H 5.69, N 3.52.

*(E)-N-(2-(10β-Dihydroartemisinoxy)ethyl)-4((4-(2-(N-(2-fluoro-5-methylphenyl)sulfamoyl)vinyl)phenoxy)methyl)benzamide* (**6d**) Light yellow solid (35% yield); m.p.: 135–137 °C; MS (ESI) *m/z*: 749.2 (M-H)^–^; IR (KBr) cm^−1^: 3412.2, 2924.6, 1603.9, 1509.1, 1343.9, 1146.1, 1025.8, 984.1, 872.7, 843.9, 597.9; ^1^H-NMR (300 MHz, DMSO-*d*_6_) *δ* 8.40 (br, 1H), 7.74 (d, *J* = 8.1 Hz, 2H), 7.60 (d, *J* = 8.4 Hz, 2H), 7.41(d, *J* = 8.2 Hz, 2H), 7.28 (s, 1H), 7.20 (m, 2H), 7.13 (m, 2H), 6.83 (d, *J* = 8.1 Hz, 2H), 5.26 (s, 1H), 4.78 (s, 2H), 4.69 (d, *J* = 3.3 Hz, 1H), 4.46 (d, *J* = 3.5 Hz, 1H), 4.39 (m, 1H), 3.82 (m, 2H), 3.52 (m, 2H), 2.34 (m, 1H), 2.19 (s, 3H), 2.10 (m, 1H), 1.94 (m, 1H), 1.66 (m, 2H), 1.52 (m, 1H), 1.38 (s, 1H), 1.25 (m, 6H), 0.80 (d, *J* = 7.3 Hz, 3H), 0.66 (d, *J* = 5.6 Hz, 3H). Anal. Calcd. for C_40_H_47_FN_2_O_9_S: C 63.98, H 6.31, N 3.73. Found: C 64.02, H 6.37, N 3.68.

*(E)-N-(2-(10β-Dihydroartemisinoxy)ethyl)-4((4-(2-(N-(2-methylphenyl)sulfamoyl)vinyl)phenoxy)methyl)benzamide* (**6e**) Light yellow solid (40% yield); m.p.: 143–145 °C; MS (ESI) *m/z*: 731.2 (M-H)^–^; IR (KBr) cm^−1^: 3397.2, 2923.2, 1603.4, 1510.1, 1341.8, 1143.7, 1027.5, 984.2, 874.6, 800.3, 594.8; ^1^H-NMR (300 MHz, DMSO-*d*_6_) *δ* 8.39 (s, 1H), 7.71 (d, *J* = 8.1 Hz, 2H), 7.61 (m, 4H), 7.34 (m, 4H), 7.15 (d, *J* = 8.2 Hz, 2H), 6.83 (d, *J* = 8.2 Hz, 2H), 5.29 (s, 1H), 4.87 (m, 1H), 4.69 (m, 2H), 3.83 (m, 2H), 3.47 (m, 2H), 2.40 (m, 2H), 2.31 (s, 1H), 2.15 (m, 1H), 2.07 (s, 3H), 1.95 (m, 2H), 1.70 (m, 2H), 1.51 (m, 1H), 1.27 (m, 4H), 1.04 (m, 2H), 0.80 (d, *J* = 7.3 Hz, 3H), 0.72 (d, *J* = 4.7 Hz, 3H). Anal. Calcd. for C_40_H_48_N_2_O_9_S: C 65.55, H 6.60, N 3.82. Found: C 65.48, H 6.57, N 3.85.

*(E)-N-(2-(10β-Dihydroartemisinoxy)ethyl)-4((4-(2-(N-(4-chlorophenyl)sulfamoyl)vinyl)phenoxy)methyl)benzamide* (**6f**). Yellow solid (39% yield); m.p.: 138–141 °C; MS (ESI) *m/z*: 751.1 (M-H)^–^; IR (KBr) cm^−1^: 3397.1, 2923.1, 1603.4, 1509.9, 1345.8, 1146.5, 1026.2, 982.5, 871.4, 800.5, 594.3; ^1^H-NMR (300 MHz, DMSO-*d*_6_) *δ* 8.39 (br, 1H), 7.72 (d, *J* = 8.2 Hz, 2H), 7.59 (d, *J* = 8.5 Hz, 2H), 7.48 (d, *J* = 13.1 Hz, 2H), 7.32 (d, *J* = 8.2 Hz, 2H), 7.28 (d, *J* = 4.6 Hz, 2H), 7.17 (d, *J* = 8.1 Hz, 2H), 6.80 (d, *J* = 8.2 Hz, 2H), 5.24 (s, 1H), 4.83 (s, 2H), 4.62 (d, *J* = 3.2 Hz, 1H), 4.41 (m, 1H), 4.30 (m, 1H), 3.81 (s, 2H), 3.60 (m, 1H), 2.33 (m, 1H), 2.08 (m, 1H), 1.93 (m, 1H), 1.61 (m, 3H), 1.47 (m, 2H), 1.26 (m, 6H), 0.78 (d, *J* = 7.2 Hz, 3H), 0.64 (d, *J* = 5.8 Hz, 3H). Anal. Calcd. for C_39_H_45_ClN_2_O_9_S: C 62.18, H 6.02, N 3.72. Found: C 62.23, H 5.98, N 3.76.

*(E)-N-(2-(10β-Dihydroartemisinoxy)ethyl)-4((4-(2-(N-(2-chlorophenyl)sulfamoyl)vinyl)phenoxy)methyl)benzamide* (**6g**). Light yellow solid (38% yield); m.p.: 147–150 °C; MS (ESI) *m/z*: 775.2 (M+Na)^+^; IR (KBr) cm^−1^: 3394.1,2925.3, 1605.5, 1499.1, 1339.3, 1144.8, 1028.8, 979.5, 881.7, 806.7, 598.2; ^1^H-NMR (300 MHz, DMSO-*d*_6_) δ 7.59 (m, 2H), 7.40 (d, *J* = 8.6 Hz, 4H), 7.32 (m, 3H), 6.94 (s, 2H), 6.79 (m, 3H), 5.62 (s, 1H), 5.35 (d, *J* = 10.3 Hz, 1H), 4.80 (s, 2H), 4.68 (d, *J* = 3.1 Hz, 1H), 3.81 (m, 2H), 3.45 (m, 2H), 2.18 (m, 1H), 2.05 (m, 2H), 1.94 (m, 2H), 1.63 (m, 2H), 1.52 (m, 2H), 1.33 (s, 3H), 0.98 (m, 2H), 0.83 (d, *J* = 7.4 Hz, 3H), 0.71 (d, *J* = 5.7 Hz, 3H). Anal. Calcd. for C_39_H_45_ClN_2_O_9_S: C 62.18, H 6.02, N 3.72. Found: C 62.12, H 5.97, N 3.79.

*(E)-N-(2-(10β-Dihydroartemisinoxy)ethyl)-4((4-(2-(N-(2,6-dichlorophenyl)sulfamoyl)vinyl)phenoxy)methyl)benzamide* (**6h**). Light yellow solid (40% yield); m.p.: 151–153 °C; MS (ESI) *m/z*: 784.9 (M-H)^–^; IR (KBr) cm^−1^: 3420.8, 2922.6, 1602.6, 1508.2, 1348.2, 1147.1, 1027.8, 984.7, 874.3, 541.5; ^1^H-NMR (300 MHz, DMSO-*d*_6_) *δ* 8.40 (br, 1H), 7.75 (d, *J* = 8.1 Hz, 1H), 7.62 (d, *J* = 8.3 Hz, 2H), 7.59 (m, 4H), 7.28 (m, 4H), 6.84 (d, *J* = 7.5 Hz, 2H), 5.26 (s, 1H), 4.80 (s, 2H), 4.69 (d, *J* = 3.3 Hz, 1H), 3.85 (m, 2H), 3.59 (m, 2H), 2.35 (m, 1H), 2.13 (m, 1H), 1.97 (m, 2H), 1.67 (m, 3H), 1.52 (m, 2H), 1.27 (s, 3H), 1.24 (s, 2H), 1.04 (m, 1H), 0.81 (d, *J* = 7.4 Hz, 3H), 0.69 (d, *J* = 4.5 Hz, 3H). Anal. Calcd. for C_39_H_44_Cl_2_N_2_O_9_S: C 59.46, H 5.63, N 3.56. Found: C 59.42, H 5.59, N 3.50.

*(E)-N-(2-(10β-Dihydroartemisinoxy)ethyl)-4((4-(2-(N-(3-methoxyphenyl)sulfamoyl)vinyl)phenoxy)methyl)benzamide* (**6i**). Light yellow solid (48% yield); m.p.: 144–145 °C; MS (ESI) *m/z*: 747.2 (M−H)^–^; IR (KBr) cm^−1^: 3392.7, 2923.7, 1603.2, 1512.6, 1340.0, 1142.2, 1025.9, 982.8, 871.7, 840.7, 596.3; ^1^H-NMR (300 MHz, DMSO-*d*_6_) δ 8.40 (br, 1H), 7.74 (d, *J* = 8.2 Hz, 2H), 7.60 (d, *J* = 8.5 Hz, 2H), 7.50 (s, 1H), 7.43 (s, 1H), 7.37 (d, *J* = 8.2 Hz, 2H), 7.32 (d, *J* = 4.6 Hz, 2H), 7.26 (d, *J* = 8.1 Hz, 2H), 6.82 (d, *J* = 8.6 Hz, 2H), 5.25 (s, 1H), 4.92 (d, *J* = 5.4 Hz, 2H), 4.68 (m, 1H), 3.95(s, 3H), 3.82 (m, 2H), 3.51(m, 2H), 2.34 (m, 2H), 2.12 (m, 2H), 2.00 (m, 2H), 1.66 (m, 2H), 1.49 (m, 1H), 1.26 (s, 3H), 1.21 (m, 3H), 0.81 (d, *J* = 7.3 Hz, 3H), 0.69 (d, *J* = 5.2 Hz, 3H). Anal. Calcd. for C_40_H_48_N_2_O_10_S: C 64.15, H 6.46, N 3.74. Found: C 64.09, H 6.41, N 3.67.

*(E)-N-(2-(10β-Dihydroartemisinoxy)ethyl)-4((4-(2-(N-(3-trifluoromethyl-4fluorophenyl)sulfamoyl)vinyl)phenoxy)methyl)benzamide* (**6j**). Yellow solid (32% yield); m.p.: 152–154 °C; MS (ESI) m/z: 803.2 (M-H)^–^; IR (KBr) cm^−1^: 3401.8, 2924.7, 1602.3, 1503.7, 1328.9, 1145.3, 1026.0, 983.9, 845.8, 646.6, 542.5; ^1^H-NMR (300 MHz, DMSO-d_6_) δ 8.40 (br, 1H), 7.81 (m, 4H), 7.63 (m, 3H), 7.47 (d, J = 15.6 Hz, 1H), 7.37 (t, J = 8.4 Hz, 2H), 7.27 (d, J = 15.6 Hz, 1H), 6.82 (d, J = 8.6 Hz, 2H), 5.24 (s, 1H), 4.98 (s, 1H), 4.93 (s, 2H), 4.68 (d, J = 3.1 Hz, 1H), 3.83 (m, 2H), 3.51 (m, 2H), 2.34 (m, 1H), 2.16 (m, 2H), 1.94 (m, 1H), 1.65 (m, 2H), 1.49 (m, 1H), 1.26 (s, 4H), 1.14 (m, 2H), 1.05 (m, 1H), 0.79 (d, J = 7.3 Hz, 3H), 0.60 (d, J = 6.0 Hz, 3H). Anal. Calcd. for C_40_H_44_F_4_N_2_O_9_S: C 59.69, H 5.51, N 3.48. Found: C 59.74, H 5.46, N 3.41.

### 3.6. Evaluation of Biological Activity

The cytotoxic activities of compounds **3a**–**h** and **6a**–**j** were evaluated with the HT-29, MDA-MB-231, U87MG, H460, A549, HL-60 cancer cell lines and one normal cell line, WI-38, by the standard MTT assay *in vitro* [20], with DHA as the positive control. The cell lines were cultured in minimum essential medium (MEM) supplement with 10% fetal bovine serum (FBS). Approximately 4 × 10^3^ cells, suspended in MEM medium, were plated on to each well of a 96-well plate and incubated in 5% CO_2_ at 37 °C for 24 h. The test compounds at indicated final concentrations were added to the culture medium and the cell cultures were continued for 72 h. Fresh MTT was added to each well at a terminal concentration of 5 μg/mL and incubated with cells at 37 °C for 4 h. The formazan crystals were dissolved in 100 μL DMSO each well, and the absorbency at 492 nm (for absorbance of MTT formazan) and 630 nm (for the reference wavelength) was measured with the ELISA reader. All of the compounds were tested twice in each of the cell lines. The results expressed as IC_50_ (inhibitory concentration 50%) were the averages of two determinations and calculated using the Bacus Laboratories Incorporated Slide Scanner (Bliss) software.

## 4. Conclusions

In summary, two series of 10-substituted dihydroartemisinin derivatives containing *N*-aryl phenylethenesulfonamide groups were prepared. Through anti-tumor activity screening the following conclusion can be reached about their structure-activity relationships: (a) all the target compounds were more potent than DHA and displayed less toxicity against the normal cell line WI-38, indicating that the presence of *N*-phenyl phenylethenesulfonamides moiety significantly enhanced their antitumor activities against the six cancer cell lines; (b) most compounds displayed good selectivity for inhibition of MDA-MB-231, HT-29, and especially the HL-60 cancer cell lines; (c) the substituent on the N-phenyl ring might play an important role in the tested compounds’ activities. The introduction of halogen, especially trifluoromethoxy group at the 4-position of the *N*-phenyl group could enhance the cytotoxic activities, while methyl or methoxy groups decreased the potency.
